# Fine Structure of the Eggshell of the Blow Fly, *Lucilia cuprina*

**DOI:** 10.1673/031.007.0901

**Published:** 2007-02-19

**Authors:** Kabkaew L. Sukontason, Nophawan Bunchu, Tarinee Chaiwong, Budsabong Kuntalue, Kom Sukontason

**Affiliations:** ^1^ Department of Parasitology, Faculty of Medicine, Chiang Mai University, Chiang Mai 50200, Thailand; ^2^ Electron Microscopy Research and Service Center (EMRSC), Faculty of Science, Chiang Mai University, Chiang Mai, Thailand

**Keywords:** eggshell, ultrastructure, *Lucilia cuprina*, forensic entomology

## Abstract

The fine structure of the eggshell of blow fly, *Lucilia cuprina* (Wiedemann) (Diptera: Calliphoridae), was examined using scanning and transmission electron microscopy. Eggs, 1.09±0.07 mm in length and 0.25±0.05 mm in width, bore a relatively wide plastron that extending along almost the entire length. The polygonal pattern of chorionic sculpture was indistinct. The ultrathin section indicated a multi-layered eggshell having an exochorion, outer endochorion, pillars, an inner endochorion, innermost chorionic layer, and a wax layer. This study provides new information about the fine morphology of blow flies eggs. A key to differentiate the eggs of forensically important flies in Thailand is given.

## Introduction

*Lucilia* (=*Phaenicia*) *cuprina* (Wiedemann, 1830) (Diptera: Calliphoridae) is a fly of medical and veterinary importance, not only as an ectoparasite, but also because it causes myiasis in humans and other mammals, particularly sheep (Zumpt 1965; Stevens and Wall 1997; Tellam et al. 2001; Colditz et al. 2002). It has recently been claimed to be forensically important, since *L*. *cuprina* was found associated with corpses and could be used in forensic investigations (Smith 1986; Goff 2000; Byrd and Castner 2001; Greenberg and Kunich 2002). In Thailand, the larvae of this species have been found in human corpses in Chiang Mai, northern Thailand (KL Sukontason, unpublished data). Systematically, *L*. *cuprina* has been classified in the Family Calliphoridae, Subfamily Calliphorinae, and Tribe Luciliini (Kurahashi et al. 1997).

The presence in corpses of fly eggs, larva or puparia, as well as other arthropods can be used in forensic investigation. For example, the presence of only fly eggs in a corpse can used to estimate a short postmortem interval (Smith 1986; Lord 1990; Anderson 1999; Byrd and Castner 2001; Anderson 2004). However, an essential first step is the species identification of fly eggs. The identity of fly eggs has been performed using light microscopy (Sukontason et al. 2004a) or scanning electron microscopy (SEM) (Kitching 1976; Greenberg and Szyska 1984; Greenberg and Singh 1995). In this study, the fine structure of the eggshell of *L*. *cuprina* is presented using SEM and transmission electron microscopy (TEM). A key is also provided to differentiate *L*. *cuprina* eggs from other forensically important fly species in Thailand.

## Materials and Methods

The eggs of *L*. *cuprina* were obtained from the laboratory colony maintained at the Department of Parasitology, Faculty of Medicine, Chiang Mai University. The rearing procedure was modified by using the technique of Haskell (1990) at room temperature (average, 24–28°C). Fresh pork liver was provided as a larval food source and oviposition site.

For the SEM process, eggs were washed several times using normal saline solution to remove any pork liver tissue residue. The specimens were fixed with 2.5% glutaraldehyde in phosphate buffer solution (PBS) at a pH of 7.4 at 4°C for 24 h. They were then rinsed twice with PBS at 10-min intervals. The rinsed eggs were then treated with 1% osmium tetroxide at room temperature for one day for post-fixation. This was followed by rinsing the eggs twice with PBS and dehydrating with increasing concentrations of alcohol as follows: 30, 50, 70, 80 and 90%. The eggs remained in each concentration of alcohol for 12 h during each step of the dehydration process. The eggs were then placed in absolute alcohol for two 12 h periods followed by acetone for two 12 h periods. Finally, the eggs were subjected to critical point drying in order to complete the dehydration process. In order to view the eggs, they were first attached to aluminum stubs with double-stick tape so they could be coated with gold in a sputter-coating apparatus before being viewed with a JEOL-JSM840A scanning electron microscope (JOEL, www.jeol.com).

The procedure for the TEM process was the same as that for SEM until the eggs were placed in absolute alcohol for two 12 h periods. After that, they were placed in acetone for 2 h before transferring to a ratio of resin:acetone 1:3 for 24 h, 1:1 for 24 h and 3:1 for 24 h, followed by resin for 2, 3 h periods. Egg specimens were embedded in Spurr's resin by placing them into a plastic block, and incubating at 70 °C for 24 h. Section of the eggs was made with a glass knife on an Ultramicrotome (Leica, www.leica-microsystems.com). The ultra-thin section was stained with uranyl acetate and lead citrate; and observed under the JEOL 1200.

To differentiate *L. cuprina* eggs from other forensically important flies, five species of flies were included in this study, namely Callliphoridae *Chrysomya megacephala*, *Chrysomya rufifacies*, and *Chrysomya nigripes*; Muscidae, *Musca domestica*, *Synthesiomyia nudiseta*; and Phoridae, *Megaselia scalaris.* The eggs were either processed by the SEM or stained with one percent of potassium permanganate solution, as previously described by Sukontason et al. 2004a. Terminology used for of describing fly eggshell followed Margaritis (1985).

## Results and Discussion

**Figure 1.**
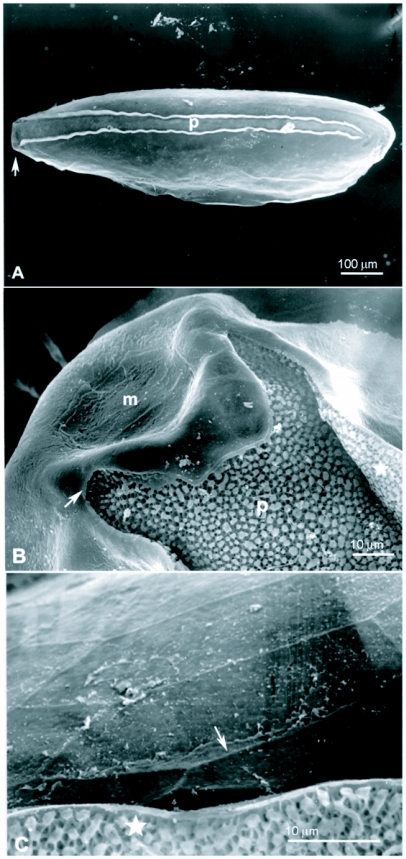
Scanning electron micrographs of egg of *L*. *cuprina.* A (Above) Whole egg showing the wide plastron region (p) extending along almost the entire length. Arrow indicates anterior end bearing the micropyle. B (Middle) Plastron (p) near micropyle (m) showing slight bifurcation. Arrow indicates the end of bifurcation; star indicates upright plastron region along the hatching line. C (Lower) Chorionic sculpture that has a smooth surface inside the indistinct polygonal patterns (arrow). Star indicates the upright plastron region along the hatching line.

**Figure 2.**
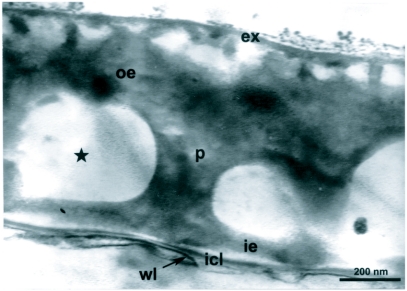
Transmission electron micrograph of the eggshell of *L*. *cuprina* showing a multi-layered surface with the outermost exochorion (ex), outer endochorion (oe), and layer of vertical pillars (p) between the irregular space of aeropyles (star), inner endochorion (ie), innermost chorionic layer (icl), wax layer (wl).

The eggs of *L. cuprina* were creamy-white, elongated and 1.09±0.07 mm in length, 0.25±0.05 mm in width (*n =* 50). The plastron originated from the anterior end near the micropyle, and extended dorsally along almost the entire length (Figure 1A). The width of the plastron was 0.022±0.006 mm (*n* = 50), representing ≈8.8% of the width of the eggs. The plastron adjacent to the micropyle was slightly bifurcated (Figure 1B), whereas that along the hatching line (Figure 1B, 1C, stars) was upright. The chorionic sculpture had a polygonal pattern (pentagonal or hexagonal) with indistinct boundary (Figure 1C, arrow). Under TEM observation, a section of the eggshell reveals a multi-layered surface with the outermost exochorion, outer endochorion, a layer of vertical pillars between the irregular space of aeropyles (star), inner endochorion, innermost chorionic layer, and wax layer (Figure 2).

**Table 1.**
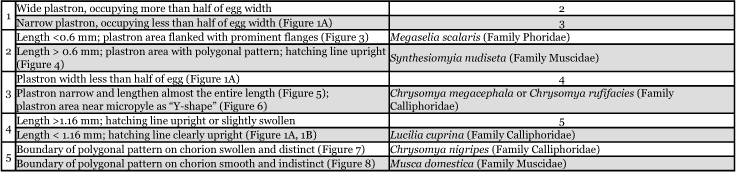
Egg identification key.

A key to simplify the identification of egg of *L*. *cuprina* from the other forensically important species in Thailand was summarized for morphological comparison as follows (Table 1).

The eggshell structure of *L*. *cuprina* conforms to general ultrastructural patterns shown by SEM micrographs of blow fly species (Kitching 1976; Erzinclioglu 1989; Liu and Greenberg 1989; Peterson and Newman 1991; Greenberg and Singh 1995; Sukontason et al. 2004b), by bearing the dorsal plastron, micropyle and polygonal pattern of chorionic sculpture. The relatively wide plastron and slight bifurcation of the plastron near the micropyle of *L*. *cuprina* eggs are similar to that found in *Lucilia* species, e.g., *Phaenicia sericata*, *Phaenicia coeruleiviridis* and *Phaenicia illustris* (Greenberg and Singh 1995). These authors indicated the difficulty in differentiating between them, however, a slight bifurcation of the plastron near the micropyle of *L*. *cuprina* differed from the marked bifurcation of *L*. *ibis* (Greenberg and Kunich 2002). Hence, this feature would be partially useful in future for differentiating eggs of the *Lucilia* species that exist in Thailand and other countries in Asia, e.g. *L. porphyrina*, *L*. *papuensis*, *L. sinensis*, *L. bismarckensis*, *L. calviceps*, *L. fumicosta*, *L*. *hainanensis* and *L*. *salazarae* (Tumrasvin et al. 1979; Kurahashi et al. 1997; Kurahashi and Magpayo 2000; Kurahashi 2001).

**Figure 3.**
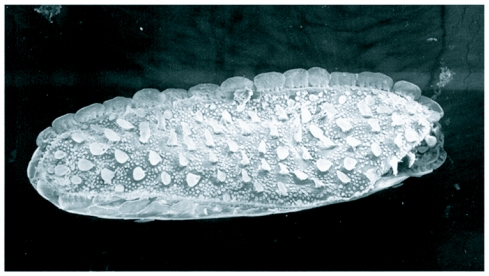
Scanning electron micrograph of egg of *M. scalaris.* Whole egg showing the wide plastron region flanked with prominent flanges.

**Figure 4.**
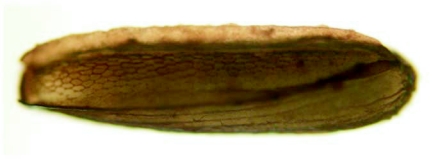
Egg of *S*. *nudiseta* after being stained with 1% potassium permanganate solution for 1 min. Whole egg showing wide plastron area with polygonal pattern and upright hatching line.

**Figure 5.**
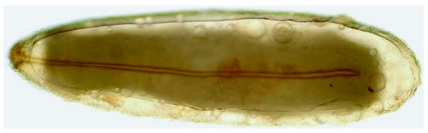
Egg of *C*. *megacephala* after being stained with 1% potassium permanganate solution for 1 min. Whole egg showing narrow plastron area lengthen almost the entire length.

**Figure 6.**
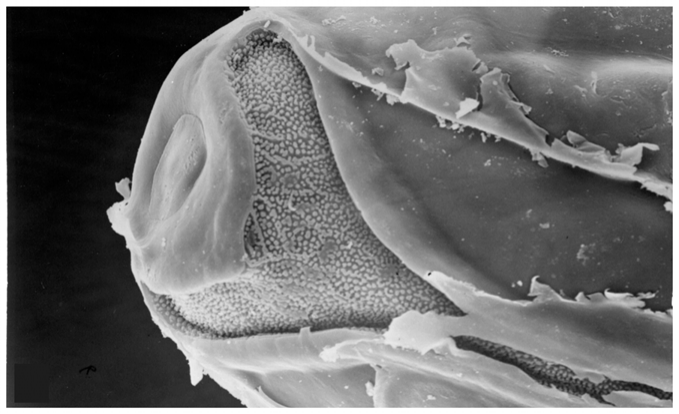
Scanning electron micrograph of egg of *C. megacephala* displaying “Y-shape” of plastron area near micropyle.

**Figure 7.**
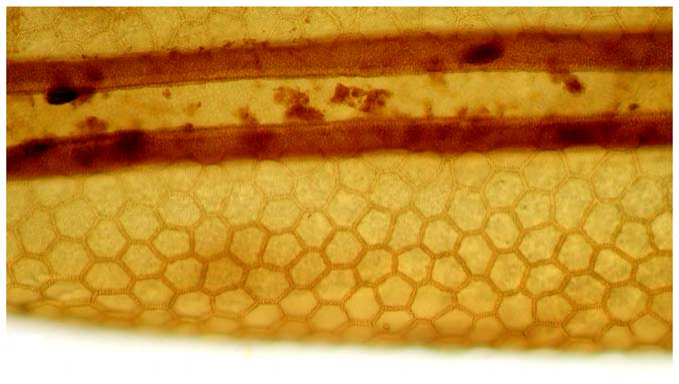
Egg of *C*. *nigripes* after being stained with 1% potassium permanganate solution for 1 min. Lower half egg showing swollen of the boundary of polygonal pattern on chorion and upright hatching line (dark brown lines).

**Figure 8.**
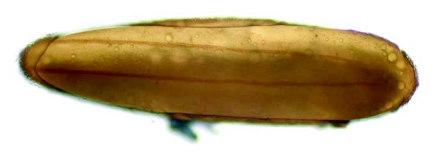
Egg of *M*. *domestica* after being stained with 1% potassium permanganate solution for 1 min. Whole egg showing wide plastron area almost the entire length and slightly swollen hatching line.

The chorionic ultrastructure has been used as one of the taxonomic characters for differentiating between fly eggs (Kitching 1976; Feliciangeli et al. 1993; Colwell et al. 1999; Sukontason et al. 2004a). The indistinct boundary of the hexagonal pattern of *L*. *cuprina* presented herein, used for incorporation with other features, may help to differentiate eggs from other *Lucilia* species.

Our TEM observation on *L. cuprina* eggs corresponds with the multi-layered eggshell of blow flies, e.g. *Lucilia sericata*, *Calliphora erythrocephala* (Hinton 1960), *Cochliomyia hominivorax* (Peterson and Newman 1991) and *Chrysomya nigripes* (Sukontason et al. 2004b). The large perforation of aeropyles in the middle layer and wide plastron enable the efficient distribution system of gases for the developing oocytes (Hinton 1960; Margaritis 1985; Ma et al. 2002).
